# Patient-acceptable symptom state for the Oxford Hip Score and Forgotten Joint Score at 3 months, 1 year, and 2 years following total hip arthroplasty: a registry-based study of 597 cases

**DOI:** 10.1080/17453674.2020.1750877

**Published:** 2020-04-22

**Authors:** Vincent P Galea, Lina Holm Ingelsrud, Isabella Florissi, David Shin, Charles R Bragdon, Henrik Malchau, Kirill Gromov, Anders Troelsen

**Affiliations:** a Harris Orthopaedic Laboratory, Massachusetts General Hospital, Boston, MA, USA;; b Department of Orthopedic Surgery, Copenhagen University Hospital Hvidovre, Hvidovre, Denmark;; c Department of Orthopaedic Surgery, Harvard Medical School, Boston, MA, USA

## Abstract

Background and purpose — Patient-acceptable symptom states (PASS) represent the level on a patient-reported outcome measure (PROM) at which patients are satisfied with postoperative outcomes. We defined the PASS for the Oxford Hip Score (OHS) and Forgotten Joint Score (FJS-12) at 3-month, 1-year, and 2-year intervals after primary total hip arthroplasty (THA).

Patients and methods — Between July 2018 and April 2019, primary THA patients in an academic medical center’s registry completed the OHS, FJS-12, and a satisfaction anchor question at 3-month (n = 230), 1-year (n = 180), or 2-year (n = 187) postoperative intervals. PASS thresholds were derived with receiver operating characteristic analysis using the 80% specificity method. 95% confidence intervals (CI) were calculated using 1,000 non-parametric bootstrap replications.

Results — 74%, 85%, and 86% of patients reported having a satisfactory symptom state at 3 months, 1, and 2 years after surgery, respectively. At 3-month, 1-year, and 2-year intervals, PASS thresholds were 34 (CI 31–36), 40 (CI 36–44), and 39 (CI 35–42) points for the OHS and 59 (CI 54–64), 68 (CI 61–75), and 69 (CI 62–75) points for the FJS-12.

Interpretation — PASS thresholds varied with time for both the OHS and the FJS-12, with lower 3-month compared with 1-year and 2-year thresholds. These PASS thresholds represent OHS and FJS-12 levels at which the average patient is satisfied with THA outcomes, helping to interpret PROMs and serving as clinically significant benchmarks and patient-centered outcomes for research.

Patient-reported outcome measures (PROMs) are commonly used to evaluate preoperative and postoperative symptom states of patients undergoing procedures such as total hip arthroplasty (THA) (Rolfson et al. 2016). Although measures such as revision or infection rates may reliably identify significant outliers in arthroplasty outcomes, the absence of such negative outcomes is not sufficient to determine whether the outcome of a procedure was satisfactory from a patient’s point of view (American Academy of Orthopedic Surgeons 2018). Within arthroplasty, there is a focus on joint-specific PROMs, but even between these PROMs there remains variation in the ways in which joint-related health is measured.

The Oxford Hip Score (OHS) and the Forgotten Joint Score (FJS-12) are 2 such PROMs. The OHS assesses hip pain and function, and has been widely used in hip arthroplasty since its development in 1996 (Dawson et al. 1998). The FJS-12, designed in 2012, is a joint-specific questionnaire that focuses on the patient’s awareness of the affected joint (Behrend et al. 2012). 3 studies comparing these 2 PROMs found a smaller ceiling effect (proportion of respondents achieving the maximum score) in the FJS-12 compared with the OHS, suggesting that the FJS-12 may be better at distinguishing between patients with good postoperative outcomes in comparison with the OHS within their respective constructs (Hamilton et al. 2016, 2017, Larsson et al. 2019).

The patient acceptable symptom state (PASS) is the threshold on a PROM most closely associated with patient satisfaction, which is assessed on a separate questionnaire (Tubach et al. 2005, Sayers et al. 2017). PASS values allow for the interpretation of PROMs within the context of a given treatment, and they may fulfil a variety of roles: as clinically significant benchmarks for procedures, as clinically relevant, patient-centered outcomes for research, and as guides for physicians to contextualize a patient’s postoperative symptom state.

Although 2 studies have presented PASS values for the OHS following THA, they have not been externally validated (Judge et al. 2012, Keurentjes et al. 2014). Furthermore, these studies did not investigate the time-dependence of the PASS. The PASS may change within the first year of surgery in accordance with changes in patient expectations during rehabilitation. 1 study established the OHS PASS 6 months after arthroplasty, while the other derived the PASS on a cohort of patients ranging between 1.5 and 6 years following THA. Another study applied a composite questionnaire-based satisfaction anchor criterion to establish an OHS value associated with patient satisfaction 1 year following THA of 37.5 points (Hamilton et al. 2018). To our knowledge, while no THA PASS values have been established for the FJS-12, a composite anchor questionnaire-based “successful treatment” anchor was used by 1 study to establish a threshold value of 74 and 70 points at 1- and 2-year intervals following THA, respectively (Rosinsky et al. 2019).

We defined PASS values for the OHS and FJS-12 at 3 months, 1 year, and 2 years following primary THA.

## Patients and methods

### Study design, patients, and data sources

**Figure 1.**
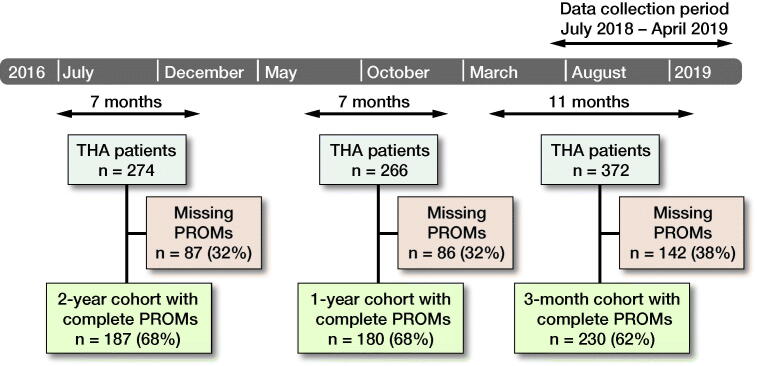
Patient selection flowchart. Patient-reported outcome measures (PROMs) were collected from patients during the data collection period from July 2018 to April 2019, and were categorized as 3-month, 1-year, and 2-year postoperative interval PROMs. Patients unable to speak or read Danish or refusing to participate in the data collection or otherwise failing to complete the PROM responses were excluded from analysis.

We performed a prospective observational cohort study analyzing data from the arthroplasty registry of a tertiary academic medical center in Denmark. Starting in March 2013, all patients undergoing primary THA due to osteoarthritis at this institution were asked to complete preoperative, 3-month, 1-year, and 2-year OHS and FJS-12 as part of the institutional registry’s data-collection process. Beginning in July 2018, all THA patients were asked to answer an additional question about satisfaction with their postoperative symptom state at each postoperative time point. These PROMs and satisfaction questions were administered electronically—patients who were unable to complete PROMs electronically were instead mailed the questionnaires. Patients were included in this study’s analysis if they had completed all of the OHS, FJS-12, and satisfaction question at any of the 3-month, 1-year, or 2-year intervals postoperatively. Patients unable to speak or read Danish, refusing to participate in the data collection, or otherwise failing to complete a PROM battery for at least 1 time point were excluded from analysis. As the satisfaction question was administered beginning in July of 2018, only PROM batteries completed between July 2018 and April 2019 were included in this analysis. Each patient and their PROMs completed during this time were subsequently categorized into 3-month, 1-year, or 2-year postoperative interval cohorts (Figure 1).

### Questionnaires

The OHS is a 12-item questionnaire that measures a patient’s pain and physical ability. Each question of the OHS allows responses ranging from 0 (worst) to 4 (best), which are scaled and summed to provide a composite score ranging from 0 (worst) to 48 (best) points (Dawson et al. 1998).

The FJS-12 is a questionnaire that assesses a patient’s awareness of his/her joint. This questionnaire was developed to assess patients’ awareness of their artificial joint following total joint arthroplasty. The PROM includes 12 questions that are each answered on a 5-level Likert scale. The FJS-12 generates a score ranging from 0 to 100 points, with a higher score indicating that the patient is less aware of the affected joint when undergoing daily activities (Behrend et al. 2012).

The satisfaction question was: *“Taking into account all the activities you have during your daily life, your level of pain, and also your functional impairment, do you consider that your current state is satisfactory?”* (Tubach et al. 2005). Possible answers are *“Yes”* and *“No.”* This question served as the PASS anchor in our derivation analyses.

### Statistics

Patient demographics and surgical variables are presented as median (interquartile range [IQR]) for non-parametric distributions, as mean (range) for parametric distributions, and as number (proportion) for categorical variables.

The correlation of the OHS and FJS-12 to the anchor question was visualized with boxplots and assessed via point-biserial coefficients.

The SPSS Statistics Version 24.0 (IBM Corp, Armonk, NY, USA) software package was used for all analyses.

### Methods of anchor-based PASS derivation

3 different methods were used to derive PASS thresholds for the OHS and for the FJS-12 at 3 months, 1 year, and 2 years postoperatively. The primary method of PASS derivation was the anchor-based 80% specificity method, which has been previously shown to be the most reliable method of PASS derivation (Aletaha et al. 2009, Kvamme et al. 2010). By this method, the PASS is the numerical value on the PROM below which 80% of dissatisfied patients are correctly identified. To derive 95% confidence intervals (CI) for these PASS values, PASS values were calculated for 1,000 non-parametric bootstrapped samples of each study subcohort, and by deriving the 2.5 and 97.5 quantiles therein (Campbell 1999).

2 additional methods of PASS derivation were performed as sensitivity analyses. The 1st of these methods is the Youden method (Youden 1950), which identifies the PASS as the coordinate on the ROC curve for which there is the highest combination of sensitivity and specificity. The 2nd of these methods is the 75th percentile method (Tubach et al. 2005), which defines the PASS as the numerical value on a PROM scale beyond which 75% of patients reported satisfaction with the outcome of their procedure.  

### Ethics, funding, and potential conflicts of interest

The institutional arthroplasty registry supplying data for this analysis was approved by the national data protection agency in Denmark, where approval from the IRB is not required for registry-based studies that exclusively examine PROMs. The study was conducted in accordance with the Declaration of Helsinki. This study was fully funded by the orthopedic departments of 2 institutions and an orthopedics research lab. The authors declare no potential conflicts of interest. 

## Results

Demographic and implant data for the cohorts at each postoperative time point are presented in Table 1. Despite being composed of different patients, the 3-month, 1-year, and 2-year postoperative patient cohorts were comparable across the demographic variables assessed.

**Table 1.** Descriptive data for the 3-month, 1-year, and 2-year cohorts. Values are count (%) unless otherwise specified

	3-month	1-year	2-year
Factor	n = 230	n = 180	n = 187
Patient demographics			
Age (years), mean (SD)	68 (11)	68 (11)	67 (11)
Female (vs. male)	140 (61)	111 (62)	118 (63)
BMI, mean (SD)	27 (5)	30 (6)	27 (5)
Severe (vs. mild/moderate) OA **^a^**	46 (20)	38 (21)	32 (17)
No/mild (vs. moderate/severe)			
systemic disease ^b^	44 (19)	32 (18)	41 (22)
Implant characteristics			
Cemented femoral component ^c^	92 (40)	68 (38)	65 (35)

**^a^** Osteoarthritis graded according to Tönnis.

**^b^** According to American Society of Anesthesiologists (ASA) Score.

**^c^** All acetabular components were uncemented across all cohorts.

OHS values, FJS-12 values, and the proportion of patients reporting a satisfactory symptom state are presented in Table 2. At 3 months postoperatively, 74% of patients reported having satisfactory symptoms and this proportion was 85% and 86% at 1 and 2 years postoperatively, respectively. The mean OHS was 39 points at 3 months, and 45 and 44 points at 1 and 2 years postoperatively, respectively. Similarly, the mean FJS-12 value increased from 71 points at 3 months to 83 and 81 points at 1 and 2 years postoperatively.

**Table 2.** Patient-reported outcomes for the 3-month, 1-year, and 2-year cohorts. Values are median (interquartile range) unless ­otherwise specified

Factor	3-month	1-year	2-year
Oxford Hip Score			
preoperative	22 (17–28)	23 (17–29)	22 (18–27)
postoperative	39 (30–43)	45 (38–48)	44 (36–47)
Forgotten Joint Score			
preoperative	15 (4–29)	17 (7–29)	17 (4–27)
postoperative	71 (50–86)	83 (58–96)	81 (55–96)
Reporting satisfactory			
symptom state, n (%) ^a^	170 (74)	153 (85)	161 (86)

**^a^** Referring to PASS transition item described in methods.

### Correlation between the PROMs and the satisfaction anchor

**Figure 2.**
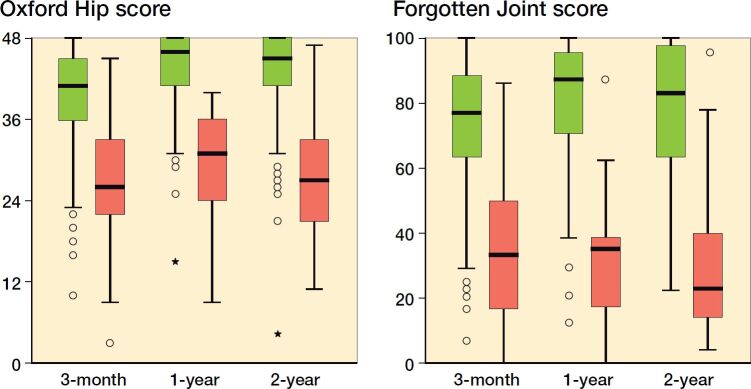
Boxplots depicting differences in Oxford Hip Scores (left panel) and Forgotten Joint Scores (right panel) between those who reported being in a satisfactory symptom state (green) and those who did not (red) for the 3-month, 1-year, and 2-year cohorts. Horisontal lines are median, boxes interquartile range (IQR), whiskers range, ● ouliers (> 1.5 x IQR), and _*_ extreme ouliers (> 3 x IQR).

The point-biserial coefficients between the OHS and the satisfaction item were 0.47 for the 3-month cohort, 0.50 for the 1-year cohort, and 0.45 for the 2-year cohort. The point-biserial coefficients between the FJS-12 and the transition item were 0.51 for the 3-month cohort, 0.53 for the 1-year cohort, and 0.56 for the 2-year cohort. FJS-12 and OHS values were lower for most patients who answered *“No”* to the satisfaction question, when compared with those who answered *“Yes”*—this held true across all time-point cohorts (Figure 2).

### PASS thresholds

**Figure 3.**
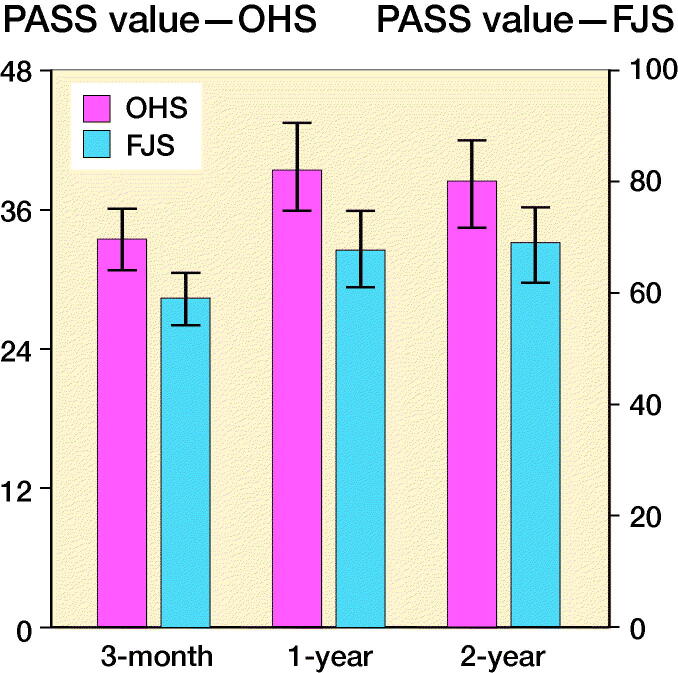
Patient acceptable symptom state (PASS) thresholds for each patient reported outcome measure at 3 months, 1 year, and 2 years after total hip arthroplasty calculated using the 80% specificity method. Error bars represent 95% confidence intervals calculated using 1,000 non-parametric bootstrap replications. Possible FJS-12 values range from 0 to 100 points, while OHS values range from 0 to 48 points.

Based on the primary method of PASS derivation, PASS threshold values were found to be 34 (CI 31–36), 40 (CI 36–44), and 39 (CI 35–42) points on the OHS and 59 (CI 54–64), 68 (CI 61–75), and 69 (CI 62–75) points on the FJS-12, for the 3-month, 1-year, and 2-year cohorts, respectively. There was minimal variation in PASS thresholds for both PROMs when comparing the 3 methods of derivation (Table 3). The 3-month PASS thresholds were observed to be lower than those of the 1-year and 2-year cohorts for both the OHS and the FJS-12 (Figure 3).

**Table 3.** PASS analysis at each follow-up interval for the Oxford Hip Score and Forgotten Joint Score using 3 different methods to determine threshold values

PROM Cohort	ROC curves AUC (95% CI)	p-value	PASS values Methods ^a^		
			A	B	C
Oxford Hip Score					
3-month	0.84 (0.77–0.90)	< 0.001	34	33	34
1-year	0.92 (0.90–0.99)	< 0.001	40	38	41
2-year	0.90 (0.83–0.98)	< 0.001	39	38	40
Forgotten Joint Score					
3-month	0.87 (0.82–0.93)	< 0.001	59	57	59
1-year	0.95 (0.90–0.99)	< 0.001	68	66	69
2-year	0.94 (0.82–0.99)	< 0.001	69	69	71

**^a^**Methods: A = 80% specificity (the primary PASS analysis method); B = Youden; C = 75th percentile.

PASS: Patient acceptable symptom state.

PROM: Patient reported outcome measure.

ROC: Receiver operating characteristic.

AUC: area under the curve.

## Discussion

We derived PASS values for the OHS, a well-established and widely used PROM, and for the FJS-12, a newer and promising PROM, in the early follow-up period after THA. PASS thresholds were 34, 40, and 39 points for the OHS and 59, 68, and 69 points for the FJS-12, for the 3 months, 1-year, and 2-year cohorts, respectively.

PROMs provide an objective way to measure a patient’s subjective experience (Gagnier 2017). The many PROMs used in orthopedics ask different questions of patients and quantify different constructs, ranging from general health to joint pain, joint function, and joint awareness. In addition to assessing distinct constructs, each PROM can be applied to evaluate treatments that have different goals and therefore different expected results. While PROMs include the patient perspective, the heterogeneity of both the available PROMs as well as their different applications complicates their interpretation. The PASS can provide valuable insight into the interpretation of PROMs for clinicians and researchers alike by identifying the value on a PROM scale at which patients consider their symptom state to be satisfactory following a given procedure. This, in turn, enables clinicians to contextualize the PROM scores of their patients, and provides researchers with a clinically relevant, patient-centered benchmark for the evaluation of surgical success.

2 previous studies have derived PASS values for the OHS at intervals of 6 months and 3 years after THA (Judge et al. 2012, Keurentjes et al. 2014). The 3-year PASS derivation suffered from a wide range of sample PROM follow-up intervals, ranging from 1.5 to 6 years—such a spread of time points is antithetical to the concept of the PASS as a time-dependent measure. Although the 3-year study also derived and compared PASS values from subsets of pre- and post-3-year PROMs, it was not able to consider PROMs collected prior to 1 year after surgery (Keurentjes et al. 2014). The PASS is likely to change within the first year of surgery as patients undergo rehabilitation. A combination of time points including those earlier than 1.5 years may better map across the typical recovery period for THA. The study deriving values at 6 months was also not able to assess the potential time-dependence of the PASS, given that only 1 time-point was studied (Judge et al. 2012). In addition, that study was limited by the use of a continuous anchor (satisfaction visual analogue scale), which was arbitrarily dichotomized at the midpoint. Our study considered 3 time intervals across the early follow-up period after THA and found that PASS values were subject to change between 3 months and 1 year. Similar results were found when comparing our 3-month PASS value with the previously derived 6-month value (35). So, too, our 1- and 2-year PASS values were found to be similar to those presented by the 3-year study (42). Hamilton et al. (2018) also established threshold values for the OHS at 1 year following THA representing “treatment success”—the authors used a composite questionnaire-based anchor, obtaining a threshold value of 37.5 points for the OHS for “success.” Furthermore, Rosinsky et al. (2019), using another composite questionnaire-based successful treatment anchor for the FJS-12, found threshold values of 74 and 70 points at 1- and 2-year intervals compared with those of this study at 68 and 69 points at 1- and 3-year intervals following THA. These similarities suggest that the PASS may be somewhat robust to variations in derivation methods and criteria.

Giesinger et al. (2019) established normative values for the PROM across the US general population, which exceeded PASS thresholds derived in this study at all postoperative time intervals, indicating that restoration of symptom states to those of the general population may not be required for patients to be satisfied with THA outcomes.

Our study is not without limitations. 1st, because of data collection constraints, our 3-month, 1-year, and 2-year cohorts comprised different patients. If we had been able to follow a single cohort of patients over time, we could have better assessed longitudinal PROM changes using paired analyses. Nevertheless, a comparison of demographic and implant variables showed the 3 cohorts to be similar. 2nd, our patients were sourced from a single Danish institution, and therefore the generalizability of our PASS values may be limited given that PROM results may vary across different international regions. However, both the OHS and the FJS-12 have been found to have comparable psychometric properties across different language versions (Paulsen et al. 2012, Harris et al. 2016, Shadid et al. 2016, Hamilton et al. 2017, Klouche et al. 2018). Furthermore, the patient demographics in our study are comparable to other registry settings (National Joint Registry 2016, American Joint Replacement Registry 2017, AOANJRR 2017). PASS values are meant to represent the result with which the average patient is likely to be satisfied. A registry-based study, including patients treated by a variety of providers with a variety of implants, is an ideal way to determine such a value. External validation of the PASS values derived in this study may prove useful for assessing the generalizability of these thresholds to a broader population of patients. Additionally, future studies might consider deriving PASS values at later time-points in order to gauge whether age-related decline influences the PASS. Given the typical age range of THA patients, extending PASS analysis up to 10 years or longer may offer valuable insight.

Our study is the first to present PASS thresholds for the FJS-12 after THA. The OHS PASS values derived were found to be similar to values presented by other studies, but they provide clearer evidence of the changes in PASS over time. We found that both OHS and FJS-12 PASS thresholds increase between 3 months and 1 year, but not between 1 and 2 years. These PASS thresholds represent the level on the OHS and FJS-12 where patients undergoing THA find their symptom state acceptable.
